# Short- and Long-Term Effects of a Scapular-Focused Exercise Protocol for Patients with Shoulder Dysfunctions—A Prospective Cohort

**DOI:** 10.3390/s21082888

**Published:** 2021-04-20

**Authors:** Cristina dos Santos, Mark A. Jones, Ricardo Matias

**Affiliations:** 1 Escola Superior Saúde—Instituto Politécnico de Setúbal, 2910-761 Setúbal, Portugal; cristina.santos@ess.ips.pt; 2 Escola Superior de Saúde do Alcoitão, 2649-506 Alcabideche, Portugal; 3 Allied Health and Human Performance, University of South Australia, Adelaide 5001, Australia; mark.jones@unisa.edu.au; 4 International Centre for Allied Health Evidence, University of South Australia, Adelaide 5001, Australia; 5 Champalimaud Research and Clinical Centre, Champalimaud Centre for the Unknown, 1400-038 Lisbon, Portugal

**Keywords:** scapula neuromuscular activity and control, rotator cuff related pain syndrome, anterior shoulder instability, scapular dyskinesis, electromyographic biofeedback

## Abstract

Current clinical practice lacks consistent evidence in the management of scapular dyskinesis. This study aims to determine the short- and long-term effects of a scapular-focused exercise protocol facilitated by real-time electromyographic biofeedback (EMGBF) on pain and function, in individuals with rotator cuff related pain syndrome (RCS) and anterior shoulder instability (ASI). One-hundred and eighty-three patients were divided into two groups (*n* = 117 RCS and *n* = 66 ASI) and guided through a structured exercise protocol, focusing on scapular dynamic control. Values of pain and function (shoulder pain and disability index (SPADI) questionnaire, complemented by the numeric pain rating scale (NPRS) and disabilities of the arm, shoulder, and hand (DASH) questionnaire) were assessed at the initial, 4-week, and 2-year follow-up and compared within and between. There were significant differences in pain and function improvement between the initial and 4-week assessments. There were no differences in the values of DASH 1st part and SPADI between the 4-week and 2-year follow-up. There were no differences between groups at the baseline and long-term, except for DASH 1st part and SPADI (*p* < 0.05). Only 29 patients (15.8%) had a recurrence episode at follow-up. These results provide valuable information on the positive results of the protocol in the short- and long-term.

## 1. Introduction

The rotator cuff related pain syndrome (RCS) [1,2] and anterior shoulder instability (ASI) are the two most prevalent shoulder dysfunctions [3,4]. They are characterized by the presence of pain [5,6,7,8,9], decreased function [5,7,9], muscle weakness [5,6,10,11,12,13], altered range of motion (ROM) [5,6,9], altered scapula neuromuscular control [12,13,14], and scapular dyskinesis [12,15,16].

Research investigating the scapular orientation and kinematics in RCS compared to asymptomatic controls concluded that no irrefutable relationship could be found between the scapula orientation and RCS [17]. However, scapular-focused stabilization and motor control exercise is promoted to address scapular dyskinesis, reduce pain [18], and restore function [11] and have been included in most studies demonstrating the benefit of exercise for RCS [19,20]. Reijneveld et al. [21] found no evidence effectiveness on a scapular-focused treatment approach in patients with RCS.

When it comes to shoulder instability, there is limited research in the management to guide therapists [7]. For traumatic instability, the current literature recommends surgical treatment [8], but for atraumatic instability, physiotherapy remains the recommended course of treatment [22] in the form of exercise to improve muscle strength and proprioception [7]. Yet, the lack of specific detail about the exercises used and the low-quality studies available is a concern [3,7,13]. Both cohort [22] and randomized controlled trials [9,23] studied the effect of specific exercise programs in patients with shoulder instability. They mostly found a significant benefit in reducing pain [9,22], increasing stability [22,23], muscle strength [9,23], ROM [23], and function [22,23]. Eshoj et al. [23] reported that a neuromuscular shoulder exercise program incorporating strength, coordination, balance, proprioception, and functional kinetic chain work was superior to the standard care exercise program emphasizing strength training to increase muscle mass in patients with traumatic shoulder instability.

Several randomized studies [5,18,24] have investigated the effects of motor control and muscle strengthening exercises in patients with RCS. Above all, they observed that scapular-focused exercise leads to higher patient-rated outcomes [18], including reduction in pain level [5,18,24] and improvement in function [5,18,24], ROM [18], and strength [18,25]. Other studies investigated the effect of scapular-focused exercises on electromyographic measures of muscle activity [18,24,26,27,28,29] and the timing of onset [30,31] with no uniformity in results [31]. Studies incorporating electromyographic biofeedback (EMGBF) to guide exercise performance also reported inconsistent findings regarding its effect. Huang et al. [27] found that the use of EMGBF improves motor control in both symptomatic and asymptomatic subjects where Juul-Kristensen et al. [24] found EMGBF made no difference to pain and function outcomes. Larsen et al. [28] proposed that individuals with subacromial impingement syndrome may benefit from incorporating EMGBF to improve the neuromuscular function.

However, to date, the scapular-focused exercise incorporated in research interventions has been quite varied with a lack of clarification about the intensity, frequency, and progression of exercises and a lack of explicit objective scapular related criteria for the success and progression of exercise [16,25]. Moreover, despite being referred to as an aid in shoulder intervention [3], the exercise programs mostly have not emphasized biofeedback as a learning strategy or an objective measure of motor control. Given the conflicting results of the value and need for scapular-focused exercise, further research is needed incorporating more explicit criteria for the administration of scapular-focused exercise before the call to abandon this intervention can be heeded.

The main objective of this study was to describe the short- and long-term effects of a scapular-focused exercise protocol supported by real-time EMGBF on the level of pain and function in individuals with shoulder dysfunctions. Additionally, scapular neuromuscular activity and control, ROM, and glenohumeral flexor and abductor isometric muscle strength (GMS) were assessed to explore the mechanisms of recovery.

It was hypothesized that:

- After 4-weeks of treatment, the protocol would lead to an amelioration in both groups in pain and function (decrease in the shoulder pain and disability index (SPADI) [32] levels with a minimal clinically important difference (MCID) ranging from 8 to 13 points) [33]; decrease in the numeric pain rating scale (NPRS) level of at least a MCID of 2.17 points [34]; and decrease in the disabilities of the arm, shoulder, and hand (DASH) levels with a MCID of 10.2 points [35].
- Primary outcome ameliorations (pain reduction and function improvement) made at the 4-week assessment would be retained at the 2-year assessment in both groups.

## 2. Materials and Methods

### 2.1. Study Design

A prospective cohort was developed to implement the scapular-focused exercise protocol, with initial, 4-week, and 2-year follow-up assessments.

### 2.2. Sample

From 213 patients recruited consecutively from an outpatient orthopaedic clinic, 183 were included and 30 unable to commit to the schedule of treatments were excluded before commencing. These 183 patients were divided into two groups according to the diagnostic categorization: RCS group (*n* = 117) and ASI group (*n* = 66). All patients had a prior consultation with an orthopaedic physician who made the diagnosis and recommended physiotherapy. The mean (±standard deviation) age for the RCS group was 41.1 (±12.2) and for the ASI group 26.7 (±10.3) years. Patient symptoms originated mostly from overuse in the RCS group (59.0%) and trauma in the ASI group (48.5%). Most patients in both groups were in the chronic stage of the condition (length of symptoms for more than 6 weeks) (81.2% for the RCS group and 71.2% for the ASI group). Sample demographics and clinical information are presented in Table 1. All patients were included based on the following criteria: 1. Age between 18 and 60 years; 2. read, write, and speak Portuguese; 3. primary complaint of shoulder pain; 4. RCS or ASI clinical diagnosis. Patients were excluded if they had: 1. Neurological symptoms [36]; 2. positive thoracic outlet syndrome (screened with Allen’s and Adson’s tests) [36,37]; 3. history of shoulder surgery or fracture [38]; 4. structural injuries confirmed by imaging (e.g., ligaments and labrum); 5. symptoms reproduced by cervical examination [37,38]; 6. unable to commit to the scheduled treatments; 7. anti-inflammatory drug use.

### 2.3. Diagnostic Criteria

For RCS classification, patients were required to have current anterolateral acromial area pain [39], pain with active shoulder elevation [38], pain with passive or isometric resisted shoulder external rotation [40,41], and at least two positive results from the Neer test [42], Hawkins test [43], and Jobe/Empty can test [44]. Despite the poor diagnostic accuracy of these tests [45], they were included as assessments of impairment clinically associated with this syndrome [2]. Patients were classified ASI if they presented with current anterior or anterosuperior shoulder pain [46], pain with passive, active or resisted shoulder movement at 90° abduction combined with external rotation, and a positive apprehension-relocation-surprise test as this continuum has demonstrated the best overall diagnostic discriminative performance [47]. All patients gave written informed consent before data collection. This research had the approval of the Ethics Committee for Research of the School of Healthcare—Setúbal Polytechnic Institute.

### 2.4. Testing Procedure

**Figure 1:**
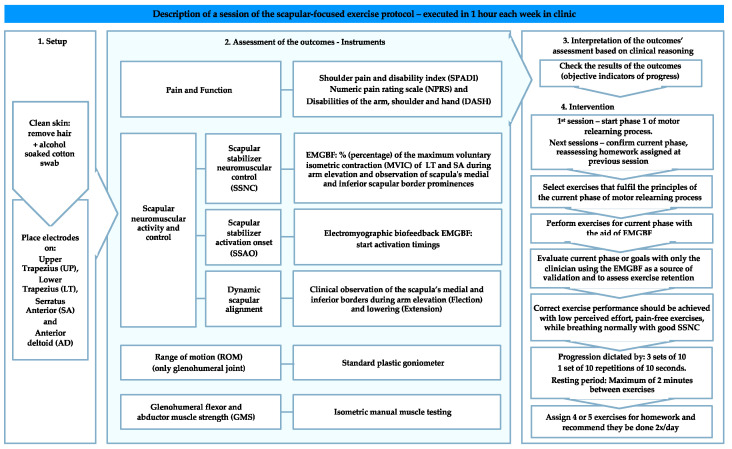
Resume of a session of the scapular-focused exercise protocol.

**Figure 2:**
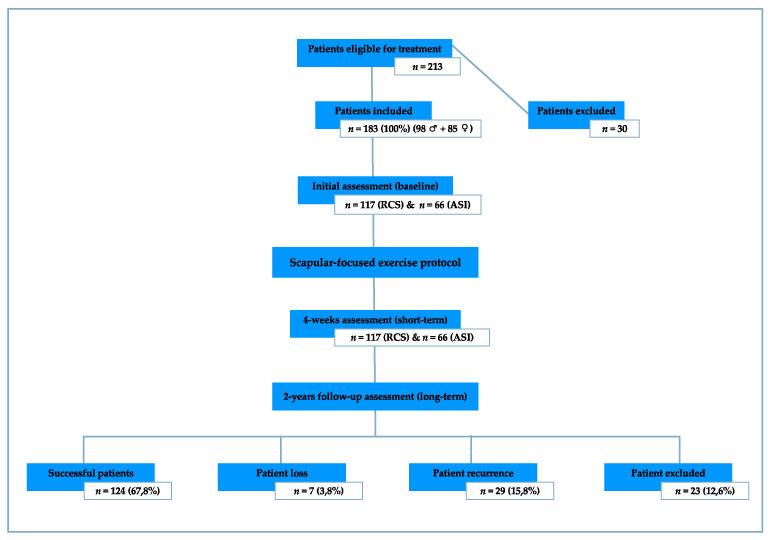
Scapular-focused exercise protocol flow diagram.

The primary outcome measure of pain and function was the SPADI [33], complemented by the NRPS [48] and DASH [35]. The secondary outcome measures of scapular neuromuscular activity and control were a combination of surface electromyography and clinical observation. The surface electromyography (Physioplux system version 1.06 comprised of four pairs of 24-mm-diameter silver chloride gel surface electrodes, a ground electrode of the same type, four electrode pair cables connected to miniaturized differential amplifiers, and a main HUB unit that communicates via Bluetooth™ to a computer) enabled both patients and the physiotherapist to assess, monitor, and correct in real-time the muscular activation and behavior during the exercises. Clinical observation of the scapula’s medial and inferior borders was used to detect scapular dyskinesis [classified as present if one or both scapular prominences (medial and inferior border) were observed during the glenohumeral movement or classified as absent if no prominence was observed [14]], using these specifications to increase the validity of the observation. Range of motion (ROM) was measured using a standard plastic goniometer (following the procedures for the glenohumeral joint motion measurements [49] recognizing the limitation of measurement without stabilization [50]). Graded glenohumeral flexors and abductors isometric muscle strength (GMS) was measured through isometric manual muscle testing (acknowledging the reduced sensitivity compared to dynamometry [51]). Outcome measures are presented in Table 2. Assessments and interventions were performed by the same examiner. All outcome assessments were carried out prior to the start of the weekly scheduled treatment (Figure 1), at 4-weeks and 2-years after the patient was discharged, hereinafter referred to as initial (baseline), 4-week (short-term), and follow-up (long-term) assessments, respectively (Figure 2).

All outcomes were assessed in the initial, weekly, 4-week, and follow-up moments as summarized in Table 2 and described in detail in the Appendix A.

### 2.5. Treatment Protocol

The treatment protocol was developed using the sequential stages of motor relearning, cognitive, associative, and autonomous [53], as a framework, while promoting the integration of local and global muscle function [54]. The treatment was divided into three phases (Appendix A) and conducted in weekly sessions to both: 1. Objectively assess the progress towards the outcomes and 2. Treat patients using exercises for the main purpose of increasing scapular neuromuscular activity and control.

### 2.6. Statistics

Descriptive statistics (means and frequency) were used to characterize the groups and variables’ distribution. The Mann-Whitney U test and Wilcoxon signed-rank test were used to compare the quantitative outcomes. Fisher’s exact test and McNemar exact test were used to compare the qualitative outcomes. Regarding the missing values (present only at follow-up) a complete-case analysis approach was adopted, assuming the missing data is completely random and unrelated to any of the variables involved in the study. The significance level was set at *p* < 0.05 and all statistical analysis was performed using the Python Software Foundation, Python Language Reference, version 3.7, available at http://www.python.org (accessed on 3 May 2020).

## 3. Results

At baseline, in the initial assessment, both RCS and ASI groups had high levels of pain and poor levels of function (SPADI, NPRS, and DASH), decreased scapular neuromuscular activity and control (SSNC, SSAO, and scapular alignment), decreased ROM and GMS. There was a difference in the scores of SPADI and DASH 1st and 3rd parts (*p* < 0.05) but none in any of the secondary outcome measures (Table 3).

After completion of the 4-weeks intervention, all outcomes improved compared with the baseline (*p* < 0.05) in both groups and the pain and function MCID values were met. Differences were found between the groups in the outcome SPADI, NPRS, and DASH 1st part at this short-term assessment (*p* < 0.05) (Table 3).

At the 2-year follow-up assessment, for the RCS group, there were no differences with the 4-week assessment in the level of SPADI, NPRS, DASH 1st and 3rd parts, SSAO, ROM, and GMS, reflecting the maintenance of the results in the long-term. However, differences were found in the DASH 2nd part, SSNC, and dynamic scapular alignment, which indicate a loss of the gains in these outcomes in the long-term (*p* < 0.05) (Table 3).

For the ASI group, there were no differences with the 4-week assessment in the level of SPADI, NPRS, DASH 1st part, SSAO, ROM, and GMS but differences were found in the DASH 2nd and 3rd parts, SSNC, and dynamic scapular alignment at the long-term (*p* < 0.05). At the 2-year follow-up, the two groups were only different in the levels of SPADI and DASH 1st part (Table 3).

At the 2-year follow-up, five (2.7%) patients were unable to return for an objective re-assessment and instead were contacted by either email or phone to answer the outcomes not requiring their presence, seven (3.8%) patients were unreachable, 29 (15.8%) patients reported returning to physiotherapy between the treatment protocol and follow-up to seek new treatment due to the same shoulder problem (recurrence), and 23 (12.6%) were not included in the 2-year follow-up as they reported having had new traumatic incidents unrelated to their treatment in the study that resulted in them seeking further health care services (e.g., shoulder surgery, fractures, muscle or tendons ruptures, etc.).

Repeated measures for time were unfeasible due to the non-normal distribution of outcomes. The power analysis by t-tests was computed for pain and function outcomes, considering the difference between two dependent means. The results obtained showed an excellent power for all variables (0.99 < d < 1.00), given the sample size of 183 participants. These results boost confidence in the outcomes reported, reinforcing the relevance of the intervention and assessment methods on the recovery efficacy of these patients, between their initial and short-time assessments and between their short-time and long-term assessments.

## 4. Discussion

In this study of the measures taken at the initial assessment, the RCS and ASI groups were different for age, and the outcome of SPADI, DASH 1st, and 3rd part, with higher mean age and higher SPADI and DASH disability scores for the RCS group. Older patients usually present with worse function levels than younger patients [25,29]. The between-group analysis, comparing the short-term results for pain and function demonstrated differences between the two groups (Table 3).

The within-group analysis, comparing results between the initial and 4-weeks (short-term) assessments, showed that clinically meaningful changes were achieved for pain and function over time in both groups. Both outcomes reached their predefined MCID and the other outcomes presented meaningful improvements. While both groups improved significantly, it was not the diagnostic category that determined the specific exercises, rather it was an assessment of the patients’ movement/control impairments. This is consistent with the view that, even when following a protocol or recommended guidelines, the management should be tailored to the patients’ pain and disability presentations rather than the hypothesized clinical diagnostic categorization [55,56].

Two years after discharge, despite a slight loss in the outcomes, the scores of SPADI, NPRS, and DASH 1st part for the ASI group and the scores of SPADI, DASH 1st, and 3rd part for the RCS group as well as the results of ROM and GMS for both groups were not different, demonstrating that the protocol of good results was not temporary. For the outcome scapular neuromuscular activity and control, only the SSAO component maintained the 4-week results through to the long-term. The SSNC and dynamic scapular alignment components presented differences.

For pain and function, the results of SPADI, NPRS, and DASH at 4-weeks were very good for both groups. NRPS at the short-term had a mean of 1.58 (±1.29) for the RCS group and 0.91 (±1.16) for the ASI group, which is better than most studies incorporating scapular exercise to treat RCS or ASI associated shoulder pain and dysfunction [9,18,23,24,25,28,29,34]. Disability improvement presented similar gains with this study compared to others (SPADI [5,26]; DASH [29,41]). These results corroborated studies that suggested a rehabilitation program incorporating motor control exercises is effective for reducing pain and disability for patients with RCS [5,18] and ASI [9,24].

The initial results of SSNC of decreased activity in LT and SA muscles corroborated the presupposition that shoulder dysfunctions comprise an alteration in the scapulothoracic stabilizer function [37], consistent with the findings of DeMey et al. [26]. Contrarily, Larsen et al. [28] reported a non-significant tendency to a higher level of mean UT, LT, and SA muscle activity in RCS patients compared to those without RCS. Collectively, these findings support the view that diagnostic categorization does not predict the muscle function, rather it is the presence of muscle dysfunction that represents either a risk variable that may contribute to pain and disability or a central nervous system response to pain and threat [57]. The initial SSNC findings in this study may reflect dysfunction in the feedforward processing present even before the onset of movement [58]. This general initial motor plan is expected to be fine-tuned using real-time internal feedback mechanisms. With a planning-control model underpinning the assessment and management of motor control/function, two principles guided the management of abnormal neuromuscular activity and motion in this study: (1) Treatment strategies to re-educate neuromuscular activity and control incorporating criteria for a preferred pattern of muscle activation prior to and during the execution of a motor command; (2) optimization of internal feedback mechanisms, so a deviation or perturbation of predicted movement can be effectively detected and corrected in real-time. Roy et al. [59,60] showed that conscious movement training with feedback causes immediate effects on motor strategies and can restore the force-couple activation in the scapular muscles, especially the stabilizers, consistent with the improvement in LT and SA activity in both groups of this study.

Concerning SSAO, half of the sample in this study already presented a feedforward mechanism [61] rather than a feedback mechanism found in other studies [36,62,63]. This highlights that the pattern of activation alone is not responsible for the patients’ symptoms and disability. This is not surprising as physical impairments, whether they are of posture, mobility, motor control or others, do not predict pain and disability [55] and motor responses to pain are variable [64]. Rather, physical impairments, in this case in SSAO, can only be judged as potential predisposing or contributing factors that may contribute to some patients’ disabilities depending on their lifestyle behaviors and requirements. Through the exercise protocol, patients who initially presented with a feedback mechanism changed to a feedforward one, as in other studies [65,66] where it is defended that the muscle pattern of onset can be improved by therapeutic exercises [65], and that the mechanisms can be trained, shifting from feedback to feedforward, while the movement is trained and repeated [66]. Contrary to these findings, DeMey et al. [26] observed no change in the recruitment timing after the treatment and Larsen et al. [28] saw no significant differences in muscle activation onset between patients with and without RCS, however neither of those studies incorporated biofeedback or motor performance criteria for facilitating learning and guiding the progression of exercise.

Contemporary neuroscience and motor control theory hold that pain alters motor patterning/control variably in response to the individual’s conscious and unconscious perception of threat, leading to changes in movement and motor function to provide protection from further pain, injury or threat [57,64,67]. Strategies that reduce pain, dysfunction and threat generally will, in turn, alter central processing, motor control, and disability [55,62,65]. As such, the reduction in the level of pain and the improvement in the level of function found in this study cannot be attributed to a single variable such as motor control. However, the approach to the scapular-focused exercise, emphasizing non-aggravating controlled progression of exercise with feedback, encouragement, and guidance in load management, likely contributed to reduced threat alongside improved control/strength leading to improvement.

The dynamic scapular alignment showed significant differences between the initial and 4-week assessments with very good results, but around 40% of the patients lost their gains at the follow-up, despite the great results of the pain and function, SSAO, ROM, and GMS outcomes. This supports the previous literature challenging the relationship between scapular alignment and RCS [1,17,68]. While a scapular-focused exercise protocol has been demonstrated in this study to be effective at reducing pain and disability; improving dynamic scapular alignment alone is not predictive of disability; and strategies to evaluate the contribution of scapular and other malalignments, such as the shoulder symptom modification procedure, described by Lewis [1], may prove helpful in predicting the potential contribution of dynamic scapular alignment to the individual patients’ pain and dysfunction. Moreover, the kinematic analysis would provide a more objective analysis of scapular alignment in any future study.

High recurrence rates are common in shoulder dysfunctions, particularly in sport activities [68]. At a 3-month follow-up, Struyf et al. [18] found maintenance of the effects of a scapular-focused treatment in patients with RCS. Given the increasing body of evidence from studies demonstrating no increased clinical benefit from surgery compared with exercise [69], it seems reasonable that patients with RCS or ASI associated shoulder pain and dysfunction should undergo a conservative trial of rehabilitation before considering surgical options. In the current study, only 29 patients (15.8%) had a recurrence episode (new symptoms due to the same problem that brought them to physiotherapy in the first place).

The results of this study support other research [13,27,40,69,70,71,72,73] that a progressive scapular-focused approach incorporating feedback and home management can significantly reduce pain and increase function in RCS and ASI associated shoulder pain. Whether the specific attention is to motor control, in particular, SSNC requires further research.

Both 1st and 2nd hypotheses were confirmed successfully with a reduction of pain and an increase of function with differences at the short-term assessment, and no differences between the short- and the long-term. Some limitations should be considered that restrict the generalizability of results: (1) No direct cause-and-effect relationship can be drawn from this protocol and these results as it did not include a control group. Further studies are needed to assess the effectiveness of this protocol against other rehabilitation approaches and clarify the contribution of EMGBF and possibly the kinematic feedback [74,75]; (2) although the diagnostic criteria reflect commonly used clinical features, the lack of gold standard diagnostic criteria compromises the RCS and ASI cohort distinctions of this study; (3) all procedures were conducted by the same researcher, although bias was minimized by the principal outcomes of pain and function being patient-rated. For the scapular neuromuscular activity and control outcome, bias was minimized by assessing SSNC and SSAO with the real-time EMGBF automatically recorded by the system. Additionally, data collection by the same researcher with extensive experience with shoulder patients and a standardized exercise approach using the EMGBF software provides consistency in procedures and measures. Both the usability and learnability of the EMGBF software and the protocol’s procedures should be assessed in the future, using a range of both novice and expert physiotherapists.

## 5. Conclusions

The presented findings suggest that a well-described scapular-focused exercise protocol, with the aid of real-time EMGBF feedback and home management, can reduce pain and increase function, as well as scapular neuromuscular activity and control, ROM, and GMS in patients with shoulder dysfunctions in the short-term. At the long-term, it appears to maintain the gains of pain and function, and the gains of SSAO, ROM, and GMS, but not for SSNC and dynamic scapular alignment. The inclusion of both ASI and RCS impairment associated groups adds evidence to the limited body of knowledge on the effect of physiotherapy on these types of shoulder dysfunctions.

## Figures and Tables

**Table 1:** Patient characteristics.

		RCS Group (*n* = 117)	ASI Group (*n* = 66)
Age (mean (SD))		41.1 (12.2)	26.7 (10.3) **
Sex (%)	Female	48 (41.0)	37 (56.1)
	Male	69 (59.0)	29 (43.9)
Origin of symptoms (%)	Trauma	30 (25.6)	32 (48.5) **
	Non-traumatic	18 (15.4)	0 (0.0) **
	Overuse	69 (59.0)	27 (40.9) **
	Sub or Dislocation	0 (0.0)	7 (10.6) **
Length of symptoms (%)	Acute (0–2 weeks)	3 (2.6)	6 (9.1)
	Sub-acute (2–6 weeks)	19 (16.2)	13 (19.7)
	Chronic (+6 weeks)	95 (81.2)	47 (71.2)
Symptomatic side (%)	Dominant	80 (68.4)	53 (80.3) *
	Non-Dominant	34 (29.0)	9 (13.6) *
	Bilateral	3 (2.6)	4 (6.1) *

Abbreviations: RCS: Rotator cuff related pain syndrome; AS: Anterior shoulder instability; SD: Standard deviation; * *p* < 0.05; ** *p* < 0.001 between-groups.

**Table 2:** Resume of testing procedure.

Outcome		Goal	Instrument	MCID	Assessment Procedures
Pain and Function		Determine pain intensity between assessment moments and measure and monitor function and symptoms over time	SPADI [32]	ranging from 8 to 13 points [33]	Filling in the SPADI questionnaire
			NPRS [48]	2.17 [34]	Patient asked to report the worst pain felt in the last week
			DASH [35]	10.2 [33]	Filling in the DASH questionnaire
Scapular neuromuscular activity and control	SSNC	Assess the muscular percentage of MVIC activity of LT, SA, and UT during arm elevation and lowering	EMGBF, Physioplux^TM^ system version 1.06	N/A	Actively raise (Flexion) then lower (Extension) the arm at a controlled self-paced velocity through maximum painless ROM in the sagittal, scapular, and frontal planes from a natural standing position for one set of three repetitions with a 20-s pause between repetitions
	SSAO	Assess muscular activation onset during rapid active shoulder elevation	EMGBF, Physioplux^TM^ system version 1.06	N/A	Actively raise (Flexion) the arm as rapidly as possible, without exacerbating pain or discomfort, to a maximum arm elevation angle of 45° in the sagittal, scapular, and frontal planes from a natural standing position for one set of three repetitions with a 20-s pause between repetitions
	Dynamic Scapular Alignment	Detect scapular dyskinesis	Clinical observation of the scapular medial and inferior border [14]	N/A	Clinical observation of the scapular medial and inferior border behavior during the arm elevation (Flexion) and lowering (Extension)
ROM		Assess glenohumeral ROM	Standard goniometer [49]	N/A	Normative ROM assessment with a standard goniometer
GMS		Assess glenohumeral flexor and abductor muscle strength	Isometric manual muscle testing [52]	N/A	Measured in a sitting position with the arm at 90° in the sagittal and frontal planes, respectively. Manual resistance was applied against the forearm with the elbow extended.

Abbreviations: MCID: Minimal Clinically Important Difference; SPAD: Shoulder pain and disability index; NPRS: Numeric pain rating scale; DASH: Disabilities of the arm, shoulder, and hand; SSNC: Scapular stabilizer neuromuscular control; MVIC: Maximum voluntary isometric contraction; LT: Lower trapezius; SA: Serratus anterior; UT: Upper trapezius; EMGBF: Electromyographic biofeedback; ROM: Range of motion; SSAO: Scapular stabilizer activation onset; GMS: Glenohumeral flexor and abductor muscle strength; N/A: Non applicable.

**Table 3:** Comparison of outcomes within groups and between-groups.

		RCS Group			ASI Group		
		Initial (*n* = 117)	4-Weeks (*n* = 117)	2-Year Follow-Up (*n* = 93)	Initial (*n* = 66)	4-Weeks (*n* = 66)	2-Year Follow-Up (*n* = 54)
SPADI (0–100)		42.07 ± 18.64	9.03 ± 8.21 **	8.62 ± 15.12	32.74 ± 19.50 ‡‡	4.80 ± 5.66 **‡‡	7.24 ± 15.78 ‡
NPRS (0–10) (Worst Pain felt)		5.85 ± 1.97	1.58± 1.29 **	1.46± 2.05	5.27 ± 2.34	0.91 ± 1.16 **‡‡	1.21 ± 1.96
DASH 1st part (0–100 point)		33.55 ± 16.53	7.63 ± 6.85 **	7.51 ± 12.92	28.47 ± 15.48 ‡	4.93 ± 5.78 **‡‡	4.37 ± 9.02 ‡
DASH 2nd part (0–100 point)		10.69 ± 19.25	2.83 ± 6.84 **	1.58 ± 7.54 *	8.60 ± 18.16	1.80 ± 4.63 **	0.22 ± 1.17 *
DASH 3rd part (0–100 point)		45.88 ± 29.01	12.50± 14.27 **	10.00± 17.59	53.80 ± 31.01 ‡	9.66 ± 12.61 **	8.15 ± 16.47 *
SSNC	Diminished (poor or moderate)	117 (100.00)	78 (66.67) **	61 (65.59) *	66 (100.00)	39 (59.09) **	22 (47.74) *
	Good	0 (0.00)	39 (33.33) **	32 (34.41) *	0(0.00)	27 (40.91) **	32 (59.26) *
SSAO (ms)	Feedback	59 (50.43)	22 (18.80) **	18 (19.35)	32 (48.48)	8 (12.12) *	7 (12.96)
	Feedforward	58 (49.57)	95 (81.20) **	75 (80.65)	34 (51.52)	58 (87.88) *	47 (87.04)
Dynamic Scapular Alignment	“YES” scapula dyskinesis (IB, MB or both prominences)	117 (100.00)	85 (72.65) **	49 (52.69) *	100 (100.00)	43 (65.15) **	32 (59.26) *
	“NO” scapula dyskinesis (no prominences)	0 (0.00)	32 (27.35) **	44 (47.31) *	0 (0.00)	23 (34.85) **	22 (40.74) *
ROM	Decreased	102 (87.18)	13 (11.11) **	9 (9.68)	51 (77.27)	1 (1.52) **	1 (1.85)
	Normal	15 (12.82)	104 (88.89) **	84 (90.32)	15(22.73)	65 (98.48) **	53 (98.15)
GMS	Decreased	114 (97.44)	30 (25.64) **	19 (20.43)	65 (98.48)	13 (19.70) **	8 (14.81)
	Normal	3 (2.56)	87 (74.36) **	74 (75.57)	1 (1.52)	53 (80.30) **	46 (85.19)

Abbreviations: RC: Rotator cuff related pain syndrome; ASI: Anterior shoulder instability; SPADI: Shoulder pain and disability index; NPRS: Numeric pain rating scale; DASH: Disabilities of the arm shoulder, and hand; DASH 1st part: Daily life activities questions; DASH 2nd part: Work optional module; DASH 3rd part: Sport/performing arts optional module; SSNC: Scapular stabilizer. Neuromuscular control; SSAO: Scapular stabilizer activation onset; IB: Inferior border of the scapula; MB: Medial border of the scapula; ROM: Range of motion; GMS: Glenohumeral flexors and abductors isometric muscle strength; * *p* < 0.05; ** *p* < 0.001 within groups; ‡ *p* < 0.05; ‡‡ *p* < 0.001 between-groups.

**Figure A1:**
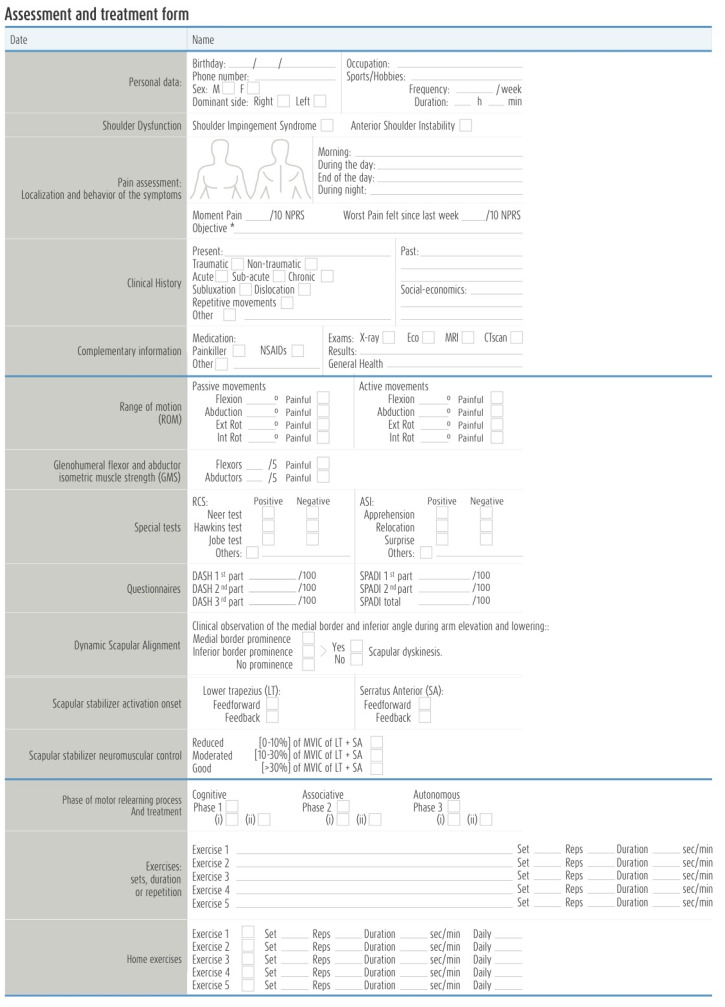
Assessment and treatment form.

**Figure A2:**
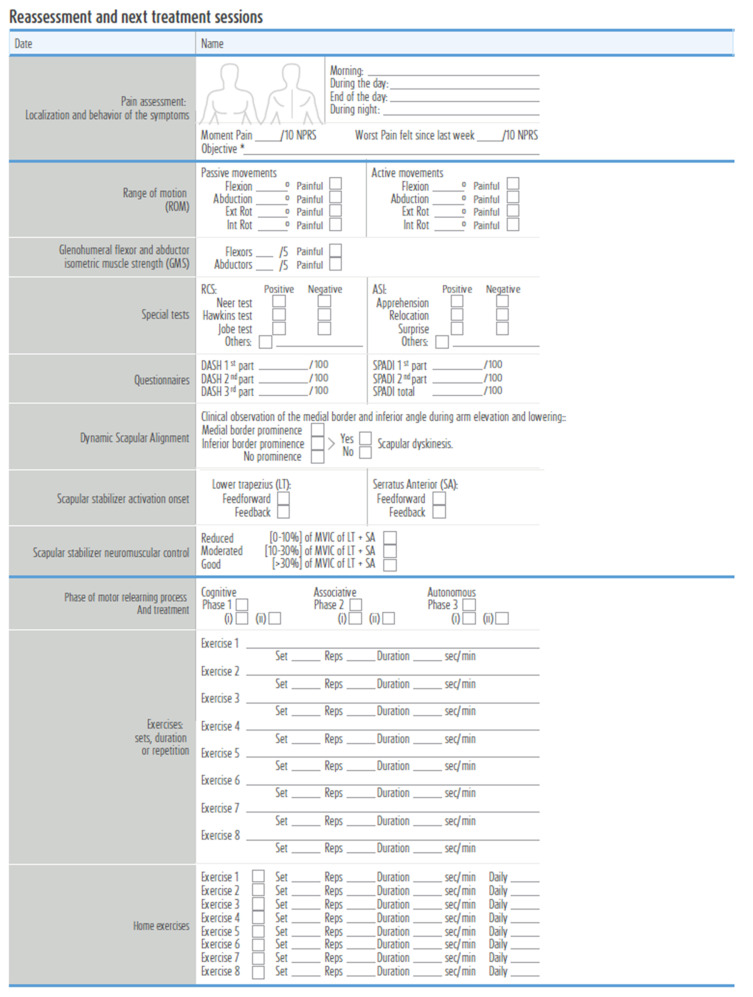
Reassessment and next treatment form.

**Figure A3:**
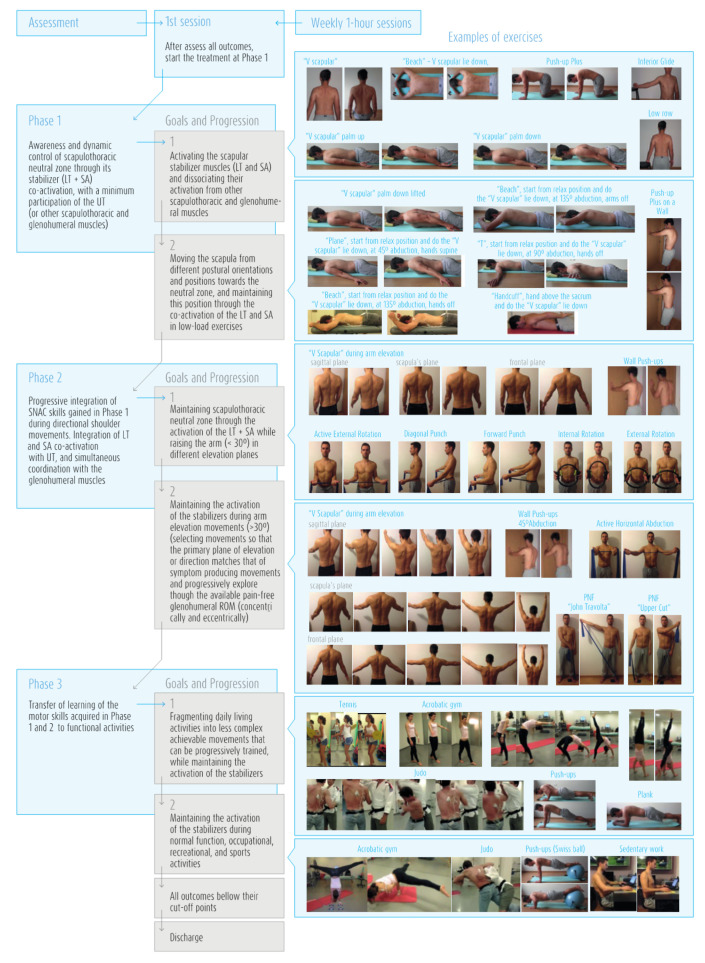
Scapular-focused treatment protocol.

## Data Availability

Individual de-identified participant data that underlie the results reported in this article will be made available to investigators whose proposed use of the data has been approved by an independent and identified review committee. Proposals should be sent to the corresponding author and requesters will need to sign a data access agreement.

## Appendix A. Detailed Description of the Scapular-Focused Exercise Protocol

In the initial, weekly, 4-week, and follow-up moments, the following assessment procedures were performed:

To assess the primary outcome of pain and function, the self-administered questionnaire shoulder pain and disability index (SPADI) [32] was used. The reported minimal clinically important difference (MCID) ranging from 8 to13 points [33] was used to determine the clinical significance of the results. To complement the assessment of this primary outcome, the numeric pain rating scale (NPRS) [48] was used to determine the pain intensity between assessment moments with a MCID of 2.17 points [34], and the self-administered questionnaire disabilities of the arm, shoulder, and hand (DASH) was used to monitor the function with a MCID of 10.2 points [33]. DASH is divided into three parts (1st: Daily life activities, 2nd: work activity, and 3rd: Sports/arts activity, respectively) [35].

To assess the scapular stabilizer activation onset (SSAO) and scapular stabilizer neuromuscular control (SSNC), the electromyographic biofeedback (EMGBF), Physioplux system version 1.06 was used. The EMGBF system was comprised of four pairs of 24-mm-diameter silver chloride gel surface electrodes, a ground electrode of the same type, four electrode pair cables connected to miniaturized differential amplifiers, and a main HUB unit that communicates via Bluetooth™ to a computer. Each amplifier had a voltage gain of 1000, input impedance higher than 100 MΩ, a common mode rejection ratio of 110 dB, and a bandwidth (−3 dB) of 25 to 500 Hz. The four amplified electromyographic (EMG) signals were then collected by the main HUB unit and converted to a digital format with a 12-bit resolution at a sampling rate of 1000 Hz. An envelope function was applied to each EMG channel using the root mean square of the mean of the absolute signal value over the last 100 milliseconds (ms). The muscle onset was determined when the EMG signal amplitude was 3 standard deviation points above the baseline signal for a 25 ms window. The baseline signal was determined by the resting EMG signal during 500 ms, collected before each activity. Prior to the surface electrode application, the patients’ skin was shaved (if necessary) and cleaned with alcohol to reduce skin impedance. The placement of the surface electrodes and the normalization of EMG data and muscle testing positions were based on the work of Ekstrom et al. [76] and Hermens et al. [77] (Table A1).

**Table A1:** Placement of the electrodes and normalization of EMG data.

Muscle	Placement of the Electrodes	Position	Normalization: Muscular Action to Measure the Maximum Voluntary Isometric Contraction
Upper Trapezius [76,77]	Between C7 spinous process and the lateral tip of the acromion	Sitting position with no back support. Shoulder abducted to 90° (no abduction in the case of pain) with the neck side-bent to the same side, rotated to the opposite side	Pressure applied to extend the head above the elbow (or to shoulder elevation in the case of pain)
Lower Trapezius [76]	At 2/3 on the line from the root of the spine of the scapula to the 8th thoracic vertebra	Sitting position with no back support Arm raised above the head in line with the lower trapezius muscle	Pressure applied against the arm elevation
Serratus Anterior [76,77]	Vertically along the mid-axillary line at the 6th rib through the 8th rib	Sitting position with no back support. Shoulder abducted to 125° in the scapular plane	Pressure applied above the elbow and at the inferior angle of the scapula attempting to de-rotate the scapula
Anterior Deltoid [76]	At one finger width distal and anterior to the acromion	Sitting position with no back support. Place the humerus in a slight external rotation to increase the effect of gravity on the anterior fibers	Pressure applied on the antero-medial surface of the arm, against abduction and flexion

All electrodes were placed over the belly, in line with the fiber directions, with an inter-electrode distance of 2.5 cm, and a ground electrode was placed over the contra-lateral clavicle. After measuring three 5-s maximum voluntary isometric contractions (MVIC) for each muscle [65] with a 20-s pause between MVIC, patients raised their arm in the sagittal plane from their standing postural natural position for two sets of three repetitions, again with a 20-s pause between repetitions. In the first set, patients were asked to perform the movement as rapidly as possible, without exacerbating pain or discomfort, to a maximum arm elevation angle of 45°, which was intended to record SSAO. The second set was performed in a controlled self-paced velocity through the patients’ maximum painless ROM both concentrically and eccentrically to assess SSNC.

SSAO can be classified as being a feedforward or feedback onset. The feedforward activation onset represents the anticipatory muscle activation that occurs prior to the mobilizer muscles and the feedback activation onset is a muscle activation that occurs after the designated feedforward period [61]. By definition, and used for this study, a feedforward activation pattern (considered as normal) was the activation of lower trapezius (LT) and serratus anterior (SA) 100 ms before to 50 ms after the anterior deltoid (AD) activation onset [61]. This outcome was computed using an accurate statistical-based method for the muscle onset detection [78]. A feedback pattern was an activation of LT and SA greater than 50 ms after AD activation [61].

SSNC levels were classified as follows: (i) Reduced when observing LT and SA activity between 0–10% of MVIC; (ii) moderate when observing LT and SA activity between 10–30% of MVIC and less than 20% of the upper trapezius (UT) MVIC activity; (iii) good when observing LT and SA activity greater than 30% of MVIC and less than 20% of UT MVIC. These levels were determined while patients concentrically flexed their arm to 90° of elevation or within their non-painful available ROM, and eccentrically returned to the initial position. The muscle MVIC percentages considered for the “good” classification were extracted from Ludewig and Cook’s [36] published results.

To assess the dynamic scapular alignment during active arm elevation and lowering, a clinical observation of the scapular medial border and the inferior angle was used to detect scapular dyskinesis. The dynamic scapular alignment was defined as normal when no prominence of the scapula medial and inferior borders was observed. The adopted dichotomous classification of scapula dyskinesis (“yes” when observing scapula medial and inferior scapular border or scapula medial border prominence or “no” when none is observed) was based on McClure et al. [14].

To assess the range of motion (ROM) a standard plastic goniometer was used and graded normal when the values corresponded with the normative ROM values expected for each movement and age group [49].

The glenohumeral flexor and abductor isometric muscle strength (GMS) was assessed by the isometric manual muscle testing [52], in a sitting position with the arm at 90° in the sagittal and frontal planes, respectively. Manual resistance was applied against the forearm with the elbow extended, graded normal (level 5 on a scale of 1 to 5) when the patient withstood the test position against a strong pressure [52], for 3 s, without losing the testing position.

Both evaluations of the outcomes and exercises intervention were recorded in the assessment, reassessment, and treatment form (Figure A1 and Figure A2):

The treatment protocol was developed using the sequential stages of motor relearning, cognitive, associative, and autonomous [53], into three phases (Table A2), as a framework, while promoting the integration of local and global muscle function [54].

**Table A2:** Motor relearning phases of the treatment protocol.

Motor Relearning Phase	Phases Description and Purpose	Progression
Phase 1	Facilitate patient pain-free awareness and dynamic control of scapulothoracic neutral zone through its stabilizers’ co-activation, namely, LT and SA, with a minimum participation of UT (or other scapulothoracic, glenohumeral, and spinal muscles)	(i) Patient should be able to activate scapular stabilizer muscles and dissociate their activation from other scapulothoracic, glenohumeral, and spinal muscles without pain provocation; (ii) Be capable of moving the scapula from different postural orientations and positions to its neutral zone (maintaining this position) through the co-activation of its scapular stabilizers in low-load exercises without pain provocation.
Phase 2	Progressively integrate scapular neuromuscular activity and control skills gained in Phase 1 during pain-free directional shoulder movements. It is currently accepted that the scapula axis of rotation changes with the increasing arm elevation and plane of movement [79]. This implies the integration of LT and SA co-activation with other scapulothoracic force generators, such as UT, and simultaneous coordination with glenohumeral muscles.	(i) Maintain scapulothoracic neutral zone by activating its stabilizers while raising (fexion) the arm (<30°) in different elevation planes, the primary aim of this stage being the focus on the scapular neuromuscular activity and control setting phase [80,81]; (ii) Arm elevation movements (>30°) should be chosen so that their primary elevation plane or direction matches that of symptom producing movements and progressively explored through the available pain-free glenohumeral ROM (concentrically and eccentrically).
Phase 3	Expected learning transfer of motor skills acquired in Phases 1 and 2 to functional activities.	(i) Fragmenting daily living activities into less complex achievable movements that can be progressively trained; (ii) and During normal function, occupational, recreational, and sports activities.

Abbreviations: LT: Lower trapezius; SA: Serratus anterior; UT: Upper trapezius; ROM: Range of motion.

The general principles for exercise prescription [82] recommend the use of variables such as the number of exercises, series, repetitions, recovery time, and the use of a periodization model [83] to support the exercise program prescription and progression. In this study, the magnitude of stimulus and progression (either in the same exercise or to progress to the next exercise or phase) were tailored to each patient’s performance and re-assessment, while operating within the protocol’s structure.

The progression guidelines were the following, as described in Table A3:

**Table A3:** Progression guidelines.

Progression Guidelines:	
Exercise complexity	Two possible sources: (i) Mechanical load, which included exercise variations that required greater arm elevation angles or the use of weights;(ii) Task or motor planning-control difficulty, which involved tasks and exercises in which it is necessary to incorporate both feedforward and feedback mechanisms of motor performance [58,84].
Feedback from the EMGBF	Provided during all sessions to facilitate the best performance at each step. However, to progress to the next exercise or phase, the patient had to demonstrate their capability to reproduce the same performance without visual feedback. At this stage, EMGBF was used by the clinician to confirm the correct exercise performance.
Perceived effort	Although a high-perceived effort is acceptable at the beginning of each phase or while increasing exercise complexity, correct exercise performance should be achieved with low perceived effort, pain-free exercise performance, and with normal breathing.
Sets, repetitions and endurance	In the absence of normative data for endurance, exercises for this population were progressed when the patient could perform three sets of 10 repetitions or hold the specified position for one set of 10 repetitions of 10 s with no pain, low perceived effort (although a high-perceived effort is acceptable at the beginning of each phase or while increasing exercise complexity), normal breathing, and good SSNC. Note, while this arbitrary performance criteria was effective for this population, the number of sets, repetitions or holding time goal for progression will vary with different patient groups according to sport, work, and lifestyle requirements.
Resting time between exercises	Although patients were encouraged to rest the least time possible between exercises, they could rest for a maximum of 2 min between exercises (especially high-loaded) but not between sets or repetitions [65].

Abbreviations: EMGBF: Electromyographic biofeedback; SSNC: Scapular stabilizer neuromuscular control.

The treatment protocol was conducted in weekly sessions to both: 1. Objectively assess the progress towards the outcomes and 2. treat patients using exercises for the main purpose of increasing scapular neuromuscular activity and control. EMGBF was used to provide patients with a real-time quality indicator for their exercise performance. SSNC was defined as a threshold (% of MVIC) of muscle activation. A minimum level of activity of LT and SA and a maximum level of activity of UT were initially set so that patients were able to achieve the objectives easily, and then thresholds were progressively increased towards their target cut-off points with a maximum step increase of 5% of MVIC. The EMGBF software was used as a form of augmented feedback to continually provide exercise performance feedback and software parameters modeled to display a green or red bar when muscle activity levels were, respectively, correctly and incorrectly attained.

At the end of each session, five homework exercises with print outs regarding sets, repetitions, and recovery time, to be completed twice daily, were assigned to the patient based upon the exercises correctly performed during the session. A schema of the scapular-focused treatment protocol, with some examples of the typical exercises executed can be seen, as follows, in Figure A3.

## References

[1] Lewis J. (2016). Rotator cuff related shoulder pain: Assessment, management and uncertainties. Man. Ther..

[2] Diercks R., Bron C., Dorrestijn O., Meskers C., Naber R., de Ruiter T., Willems J., Winters J., van der Woude H.J. (2014). Guideline for diagnosis and treatment of subacromial pain syndrome. Acta Orthop..

[3] Gibson K., Growse A., Korda L., Wray E., MacDermid J.C. (2004). The effectiveness of rehabilitation for nonoperative management of shoulder instability: A systematic review. J. Hand Ther..

[4] Michener L., McClure P., Karduna A. (2003). Anatomical and biomechanical mechanisms of subacromial impingement syndrome. Clin. Biomech..

[5] Bae Y., Lee G., Shin W. (2011). Effect of motor control and strengthening exercises on pain, function, strength and the range of motion of patients with shoulder impingement syndrome. J. Phys. Ther. Sci..

[6] Lee S., Savin D., Shah N., Bronsnick D., Goldberg B. (2015). Scapular Winging: Evaluation and Trearment. J. Bone Joint Surg. Am..

[7] Bateman M., Smith B., Osborne S., Wilkes S. (2015). Physiotherapy treatment for atraumatic recurrent shoulder instability: Early results of a specific exercise protocol using pathology-specific outcome measures. Shoulder Elbow.

[8] Friedman L., Lafosse L., Garrigues G. (2019). Global Perspectives on Management of Shoulder Instability: Decision Making and Treatment. Orthop. Clin. N. Am..

[9] Warby S., Ford J., Hahne A., Watson L., Balster S., Lenssen R., Pizzari T. (2018). Comparison of 2 Exercise Rehabilitation Programs for Multidirectional Instability of the Glenohumeral Joint: A Randomized Controlled Trial. Am. J. Sports Med..

[10] Galace De Freitas D., Marcondes F., Monteiro R., Gonçalves Rosa S., de Moraes Barros Fucs P.M., Yukio Fukuda T. (2014). Pulsed electromagnetic field and exercises in patients with shoulder impingement syndrome: A randomized, double-blind, placebo-controlled clinical trial. Arch. Phys. Med. Rehabil..

[11] Beaudreuil J., Lasbleiz S., Richette P., Seguin G., Rastel C., Aout M., Vicaut E., Cohen-Solal M., Lioté F., de Vernejoul M.-C. (2011). Assessment of dynamic humeral centering in shoulder pain with impingement syndrome: A randomised clinical trial. Ann. Rheum. Dis..

[12] Cools A., Borms D., Castelein B., Vanderstukken F., Johansson F.R. (2016). Evidence-based rehabilitation of athletes with glenohumeral instability. Knee Surg. Sports Traumatol. Arthrosc..

[13] Warby S., Pizzari T., Ford J., Hahne A.J., Watson L. (2014). The effect of exercise-based management for multidirectional instability of the glenohumeral joint: A systematic review. J. Shoulder Elbow Surg..

[14] McClure P., Greenberg E., Kareha S. (2012). Evaluation and management of scapular dysfunction. Sports Med. Arthrosc..

[15] Kibler W., Ludewig P., McClure P., Uhl T.L., Sciascia A. (2009). Scapular Summit 2009: Introduction, 16 July 16, 2009, Lexington, Kentucky. J. Orthop. Sports Phys. Ther..

[16] Kibler W., Ludewig P., McClure P., Michener L.A., Bak K., Sciascia A.D. (2013). Clinical implications of scapular dyskinesis in shoulder injury: The 2013 consensus statement from the 'scapular summit'. Br. J. Sports Med..

[17] Ratcliffe E., Pickering S., McLean S., Lewis J. (2014). Is there a relationship between subacromial impingement syndrome and scapular orientation? A systematic review. Br. J. Sports Med..

[18] Struyf F., Nijs J., Mollekens S., Jeurissen I., Truijen S., Mottram S., Meeusen R. (2013). Scapular-focused treatment in patients with shoulder impingement syndrome: A randomized clinical trial. Clin. Rheumatol..

[19] Haahr J.P., Østergaard S., Dalsgaard J., Norup K., Frost P., Lausen S., Holm E.A., Andersen J.H. (2005). Exercises versus arthroscopic decompression in patients with subacromial impingement: A randomised, controlled study in 90 cases with a one year follow up. Ann. Rheum. Dis..

[20] Holmgren T., Oberg B., Sjöberg I., Johansson K. (2012). Supervised strengthening exercises versus home-based movement exercises after arthroscopic acromioplasty: A randomized clinical trial. J. Rehabil. Med..

[21] Reijneveld E., Noten S., Michener L., Cools A., Struyf F. (2017). Clinical outcomes of scapular-focused treatment in patients with subacromial pain syndrome: Systematic review. Br. J. Sports Med..

[22] Bateman M., Osborne S., Smith B. (2019). Physiotherapy treatment for atraumatic recurrent shoulder instability: Updated results of the Derby Shoulder Instability Rehabilitation Programme. J. Arthrosc. Joint Surg..

[23] Eshoj H., Rasmussen S., Frich L., Hvass I., Christensen R., Boyle E., Lund Jensen S., Søndergaard J., Søgaard K., Juul-Kristensen B. (2020). Neuromuscular Exercises Improve Shoulder Function More Than Standard Care Exercises in Patients With a Traumatic Anterior Shoulder Dislocation. Orthop. J. Sports Med..

[24] Juul-Kristensen B., Larsen C.M., Eshoj H., Clemmensen T., Hansen A., Bo Jensen P., Boyle E., Søgaard K. (2019). Positive effects of neuromuscular shoulder exercises with or without EMG-biofeedback, on pain and function in participants with subacromial pain syndrome—A randomised controlled trial. J. Electromyogr. Kinesiol..

[25] Baskurt Z., Baskurt F., Gelecek N., Özkan M.H. (2011). The effectiveness of scapular stabilization exercise in the patients with subacromial impingement syndrome. J. Back Musculoskelet. Rehabil..

[26] DeMey K., Danneels L., Cagnie B., Cools A. (2012). Scapular muscle rehabilitation exercises in overhead athletes with impingement symptoms: Effect of a 6-week training program on muscle recruitment and functional outcome. Am. J. Sports Med..

[27] Huang H.Y., Lin J.J., Leon Guo Y., Wang W.T., Chen Y.-J. (2013). EMG biofeedback effectiveness to alter muscle activity pattern and scapular kinematics in subjects with and without shoulder impingement. J. Electromyogr. Kinesiol..

[28] Larsen C., Søgaard K., Chreiteh S., Holtermann A., Juul-Kristensen B. (2013). Neuromuscular control of scapula muscles during a voluntary task in subjects with subacromial impingement syndrome. A case-control study. J. Electromyogr. Kinesiol..

[29] Worsley P., Warner M., Mottram S., Gadola S., Veeger H.E.J., Hermens H., Morrissey D., Little P., Cooper C., Carr A. (2013). Motor control retraining exercises for shoulder impingement: Effects on function, muscle activation, and biomechanics in young adults. J. Shoulder Elbow Surg..

[30] Dorrestijn O., Stevens M., Winters J., van der Meer K., Diercks R.L. (2009). Conservative or surgical treatment for subacromial impingement syndrome? A systematic review. J. Shoulder Elbow Surg..

[31] Struyf F., Cagnie B., Cools A., Baert I., Van Brempt J., Struyf P., Meeus M. (2014). Scapulathoracic muscle activity and recruitment timing in patients with shoulder impingement symptoms and glenohumeral instability. J. Electomiogr. Kinesiol..

[32] Roach K., Budiman-Mak E., Songsiridej N., Lertratanakul Y. (1991). Development of a Shoulder Pain and Disability Index. Arthritis Care Res..

[33] Roy J., MacDermid J., Woodhouse L. (2009). Measuring Shoulder Function: A Systematic Review of Four Questionnaires. Arthritid. Rheum..

[34] Michener L., Snyder A., Leggin B. (2011). Responsiveness of the numeric pain rating scale in patients with shoulder pain and the effect of surgical status. J. Sports Rehabil..

[35] Hudak P., Amadio P., Bombardier C. (2011). Development of an Upper Extremity Outcome Measure: The DASH (Disabilities of the Arm, Shoulder and Hand)[Corrected]. The Upper Extremity Collaborative Group (UECG). J. Sports Rehabil..

[36] Ludewig P., Cook T. (2000). Alterations in shoulder kinematics and associated muscle activity in people with symptoms of shoulder impingement. Phys. Ther..

[37] Lewis L. (2009). Rotator cuff tendinopathy/subacromial impingement syndrome: Is it time for a new method of assessment?. Br. J. Sports Med..

[38] Hung C.-J., Jan M.-H., Lin Y.-F., Wang T.-Q., Liu J.-J. (2010). Scapular kinematics and impairment features for classifying patients with subacromial impingement syndrome. Man. Ther..

[39] Dong W., Goost H., Xang-Bo L., Burger C., Paul C., Wang Z.-L., Zhang T.-Y., Jiang Z.-C., Welle K., Kabir K. (2015). Treatment for Shoulder Impingement Syndrome. A PRISMA Systematic Review and Network Meta-Analysis. Medicine.

[40] Haik M., Alburquerque-Sendín F., Silva C., Siqueira-Junior A.L., Ribeiro I.L., Camargo P.R. (2014). Scapular-kinematics pre-and post-thoracic thrust manipulation in individuals with and without shoulder impingement symptoms: A randomised controlled study. J. Orthop. Sports Phys. Ther..

[41] Tate A., McClure P., Young I., Salvatori R., Michener L.A. (2010). Comprehensive impairment-based exercise and manual therapy intervention for patients with subacromial impingement syndrome: A case series. J. Orthop. Sports Phys. Ther..

[42] Neer C. (1972). Anterior acromioplasty for the chronic impingement syndrome in the shoulder: A preliminary report. J. Bone Joint Surg. Am..

[43] Hawkins R., Kennedy J. (1980). Impingement syndrome in athletes. Am. J. Sports Med..

[44] Jobe F.W., Moynes D. (1982). Delineation of diagnostic criteria and a rehabilitation program for rotator cuff injuries. Am. J. Sports Med..

[45] Hegedus E.J., Goode A., Campbell S., Morin A., Tamaddoni M., Moorman C.T., Cook C. (2008). Physical examination tests of the shoulder: A systematic review with meta-analysis of individual tests. Br. J. Sports Med..

[46] Hayes K., Callanan M., Walton J., Paxinos A., Murrell G.A.C. (2002). Shoulder instability: Management and rehabilitation. J. Orthop. Sports Phys. Ther..

[47] Hegedus E.J., Goode A., Cook C., Michener L., Myer C.A., Myer D.M., Wright A.A. (2012). Which physical examination tests provide clinicians with the most value when examining the shoulder? Update of a systematic review with meta-analysis of individual tests. Br. J. Sports Med..

[48] Huskisson E. (1982). Measurement of pain. J. Rheumatol..

[49] Norkin C., White D. (2009). The shoulder. Measurement of Joint Motion: A Guide to Goniometry.

[50] Kolber M., Hanney W. (2012). The reliability and concurrent validity of shoulder mobility measurement using a digital inclinometer and goniometer: A technical report. Int. J. Sports Phys. Ther..

[51] Celik D., Dirican A., Baltaci G., Layman J. (2012). Intrarater reliability of assessing strength of the shoulder and scapular muscles. J. Sport Rehabil..

[52] Kendall F., McCreary E., Provance P. (1993). Upper extremity and shoulder girdle strength tests. Muscles: Testing and Function, with Posture and Pain.

[53] Shumway-Cook A., Woolacott J. (2001). Motor learning and recovery of function. Motor Control: Theory and Practical Applications.

[54] Comerford M., Mottram S. (2001). Functional stability re-training: Principles and strategies for managing mechanical dysfunction. Man. Ther..

[55] Jones M.A., Rivett D.A. (2019). Clinical Reasoning: Fast and Slow Thinking in Musculoskeletal Practice. Clinical Reasoning in Musculoskeletal Practice.

[56] Salamh P., Lewis J. (2020). It is time to put special tests for rotator cuff-related shoulder pain out to posture. J. Orthop. Sports Phys. Ther..

[57] Hodges P. (2011). Pain and motor control: From the laboratory to rehabilitation. J. Electromyogr. Kinesiol..

[58] Glover S. (2004). Separate visual representations in the planning and control of action. Behav. Brain Sci..

[59] Roy J., Moffet H., Hébert L., Lirette R. (2009). Effect of motor control and strengthening exercises on shoulder function in persons with impingement syndrome: A single-subject study design. Man. Ther..

[60] Roy J., Moffet H., McFadyen B., Lirette R. (2009). Impact of movement training on upper limb motor strategies in persons with shoulder impingement syndrome. Sports Med. Arthrosc. Rehabil. Ther. Technol..

[61] Aurin A., Latash M. (1995). Directional specificity of postural muscles in feed-forward postural reactions during fast voluntary arm movements. Exp. Brain Res..

[62] Ludewig P., Reynolds J. (2009). The association of scapular kinematics and glenohumeral joint pathologies. J. Orthop. Sports Phys. Ther..

[63] Moraes G., Faria C., Teixeira-Salmela L. (2008). Scapular muscle recruitment patterns and isokinetic strength ratios of the shoulder rotator muscles in individuals with and without impingement syndrome. J. Shoulder Elbow Surg..

[64] Hodges P.W., Tucker K. (2011). Moving differently in pain: A new theory to explain the adaptation to pain. Pain.

[65] Crow J., Pizzari J., Buttifani D. (2011). Muscle onset can be improved by therapeutic exercise: A systematic review. Phys. Ther. Sport.

[66] Tsao H., Hodges P. (2008). Persistence of improvements in postural strategies following motor control training in people with recurrent low back pain. J. Electromyogr. Kinesiol..

[67] Hodges P.W., van Dillen L.R., McGill S., Brumagne S., Hides J.A., Moseley G.L. (2013). Integrated clinical approach to motor control interventions in low back and pelvic pain. Spinal Control: The Rehabilitation of Back Pain.

[68] Berkovitch Y., Shapira J., Haddad M., Keren Y., Rosenberg N. (2013). Current clinical trends in first time traumatic anterior shoulder dislocation. Merit. Res. J. Med. Med. Sci..

[69] Lewis J. (2011). Subacromial impingement syndrome: A musculoskeletal condition or a clinical illusion?. Phys. Ther. Rev..

[70] Steuri R., Sattelmayer M., Elsig S. (2017). Effectiveness of conservative interventions including exercise, manual therapy and medical management in adults with shoulder impingement: A systematic review and meta-analysis of RCTs. Br. J. Sports Med..

[71] Hanrraty C., McVeigh J., Kerr D., Basford J.R., Finch M.B., Pendleton A., Sim J. (2012). The effectiveness of physiotherapy exercises in subacromial impingement syndrome: A systematic review and meta-analysis. Semin. Arthritis. Rheum..

[72] Haik M., Alburquerque-Sendín F., Moreira R., Pires E.D., Camargo P.R. (2016). Effectiveness of physical therapy treatment of clearly defined subacromial pain: A systematic review of randomised controlled trials. Br. J. Sports Med..

[73] Bury J., West M., Chamorro-Moriana G., Littlewood C. (2016). Effectiveness of scapula-focused approaches in patients with rotator cuff related shoulder pain: A systematic review and meta-analysis. Man. Ther..

[74] Matias R., Jones M. (2019). Incorporating Biomechanical Data in the Analysis of a University Student With Shoulder Pain and Scapula Dyskinesis. Clinical Reasoning in Musculoskeletal Practice.

[75] Ferreira A.L., dos Santos C., Matias R. (2016). A kinematic biofeedback-assisted scapular-focused intervention reduces pain, and improves functioning and scapular dynamic control in patients with shoulder dysfunction. Gait Posture.

[76] Ekstrom R., Soderberg G., Donatelli R. (2005). Normalization procedures using maximum voluntary isometric contractions for the serratus anterior and trapezius muscles during surface EMG analysis. J. Electromyogr. Kinesiol..

[77] Hermens H., Freriks B., Merletti R., Stegeman D., Blok J., Rau G., Disselhorst-Klug C., Hagg G. (1999). Eurpoean Recommendations for Surface Electromyography—Results of the Seniam Project. SENIAM 8.

[78] Gamboa H., Matias R., Araújo T., Veloso A. (2012). Electromyography onset detection: New methodology. J. Biomech..

[79] Bagg S., Forrest W. (1986). Electromyographic study of the scapular rotators during arm abduction in the scapular plane. Am. J. Phys. Med..

[80] Borsa P., Timmons M., Sauers E. (2003). Scapular-positioning patterns during humeral elevation in unimpaired shoulders. J. Athl. Train..

[81] Inman V., Sauders J., Abbott L. (1996). Observations of the function of the shoulder joint. Clin. Orthop. Relat. Res..

[82] Pescatello L., Arena R., Riebe D., Thompson P. (2014). Behavioral Theories and Strategies for promoting exercise. ACSM's Guidelines for Exercise Testing and Prescription.

[83] Baechle T., Earle R. (2000). Training variation: Periodization. Essentials of Strength Training and Conditioning.

[84] Desmurget M., Grafton S. (2000). Forward modeling allows feedback control for fast reaching movements. Trends Cogn. Sci..

